# Novel function of cytoplasmic p53 at the interface between mitochondria and the endoplasmic reticulum

**DOI:** 10.1038/cddis.2015.70

**Published:** 2015-03-19

**Authors:** G Kroemer, J M Bravo-San Pedro, L Galluzzi

**Affiliations:** 1 Equipe 11 labellisée Ligue contre le Cancer, Centre de Recherche des Cordeliers, Paris, France; 2 INSERM, U1138, Paris, France; 3 Université Paris Descartes/Paris V, Sorbonne Paris Cité, Paris, France; 4 Université Pierre et Marie Curie/Paris VI, Paris, France; 5 Metabolomics and Cell Biology Platforms, Gustave Roussy Cancer Campus, Paris, France; 6 Pôle de Biologie, Hôpital Européen Georges Pompidou, AP-HP, Paris, France; 7 Gustave Roussy Cancer Campus, Villejuif, France

Germline mutations in the gene coding for tumor protein p53 (TP53, best known as p53) are associated with the Li-Fraumeni syndrome, a dominant hereditary disorder characterized by an increased predisposition of patients to the development of various tumors relatively early in life. In addition, *TP53* is affected by somatic loss-of-function mutations in a large fraction (>50%, according to current estimates) of human cancers all confounded.^1^ Finally, several other molecular defects causally associated with malignant transformation or tumor progression result in the functional inactivation of the p53 system. As a notable example, multiple neoplasms express increased levels of MDM2 proto-oncogene, E3 ubiquitin protein ligase (MDM2), resulting in an accrued degradation of p53 by the proteasome.^2^ These observations indicate that the loss of p53 functions favors the establishment and/or progression of various malignancies.

Throughout the past 25 years, p53 has been the subject of intense investigation, revealing a wide panel of mechanisms by which this protein exerts robust oncosuppressive functions.^3^ Initially, p53 was recognized for its ability to respond to DNA damage by transactivating several genes that regulate cell cycle progression (e.g., *CDKN1A*) and apoptotic cell death (e.g., *BAX*), hence preventing the propagation of potentially transforming genetic defects.^4^ Later, stress-activated p53 turned out to participate in the activation of mitochondrial apoptosis by physically interacting with pro- and antiapoptotic members of the Bcl-2 protein family (such as BAX, BCL-2 and BCL-X_L_), thereby favoring the elimination of potentially dangerous cells via transcription-independent mechanisms.^5^ More recently, several studies demonstrated that p53 mediates oncosuppressive effects not only when cells are confronted with sources of stress, but also in physiological conditions. In particular, p53 has been attributed with a key role in the preservation of physiological bioenergetic metabolism, mostly linked to its capacity to regulate the transcription of some metabolic enzymes.^6, 7^ Now, the research group lead by Paolo Pinton (University of Ferrara; Ferrara, Italy) identified yet another mechanism by which p53 mediates transcription-independent oncosuppressive functions (Figure 1).^8, 9^ In particular, Giorgi and colleagues demonstrated that cytoplasmic p53 physically stimulates the accumulation of Ca^2+^ ions within the endoplasmic reticulum (ER) by physically interacting with ATPase, Ca++ transporting, cardiac muscle, fast twitch 1 (ATP2A, best known as SERCA). This increases the efficiency of the transfer of Ca^2+^ ions between the ER and mitochondria, augmenting the propensity of (pre)malignant cells exposed to oncogenic or chemotherapeutic stress to succumb to apoptosis.^8, 9^

Previous results from the same group indicated that the extranuclear pools of several oncosuppressor proteins preferentially localize to the so-called mitochondria-associated ER membranes (MAMs),^10, 11^ which are sites of physical and functional connection between the ER and the mitochondrial network. Thus, Giorgi and co-workers set out to study the localization of cytoplasmic p53 in human colorectal carcinoma HCT 116 cells exposed to doxorubicin (an immunogenic DNA-damaging agent routinely employed for the treatment of various cancers),^12, 13^ or oxidative stress (as induced by the administration of hydrogen peroxide).^14, 15^ In line with precedent works, p53 accumulated in the cytoplasm of HCT 116 cells treated with doxorubicin or hydrogen peroxide, a phenomenon that was particularly evident when the ER and MAMs were studied as subcytoplasmic fractions.^9^ Of note, the ability of a nonlethal dose of doxorubicin to promote the accumulation of p53 at the ER was sufficient to render *Trp53*^+/+^ mouse embryonic fibroblasts (MEFs) completely sensitive to a partially lethal dose of hydrogen peroxide. Conversely, *Trp53*^−/−^ MEFs were totally resistant to hydrogen peroxide-induced cell death, irrespective of the preadministration of doxorubicin.^9^ These data confirmed the role of p53 in apoptosis induced by oxidative stress while highlighting a potential implication of the ER in this process.

Given the implication of Ca^2+^ fluxes between the ER and mitochondria in the control of mitochondrial apoptosis,^16, 17^ Giorgi and colleagues investigated the effects of the absence of p53 on reticular Ca^2+^ homeostasis. They found that *Trp53*^*-/-*^ MEFs exhibit lower steady-state reticular Ca^2+^ levels than their *Trp53*^+/+^counterparts, resulting in decreased Ca^2+^ mobilization and mitochondrial accumulation in response to ATP (a purinergic receptor agonist that is commonly employed to trigger cytosolic Ca^2+^ waves) or hydrogen peroxide. Similar results were obtained with HCT 116 cells, p53-overexpressing human cervical carcinoma HeLa cells and *Trp53*^−/−^ MEFs reconstituted with wild-type p53. Moreover, *Trp53*^+/+^ MEFs, but not their *Trp53*^−/−^ counterparts, responded to hydrogen peroxide with a fragmentation of the mitochondrial network, a phenomenon that could be drastically exacerbated by the preadministration of doxorubicin (which *per se* failed to do so).^9^ Importantly, the authors excluded the involvement of the transcriptional activity of p53 in this process by several experimental strategies, including (1) the pharmacological blockade of transcription with *α*-amanitin, alone or combined with the p53 inhibitor pifithrin *α*, to *Trp53*^+/+^ MEFs; (2) the reconstitution of *Trp53*^−/−^ MEFs with p53 mutants lacking the nuclear localization signal (NLS); and (3) the reconstitution of *Trp53*^*-/-*^ MEFs with an NLS-deficient p53 variant specifically addressed to the ER. Moreover, Giorgi and colleagues demonstrated that various naturally occurring p53 mutants, such as p53^R175H^ and p53^R273H^, are unable to restore reticular Ca^2+^ homeostasis in *Trp53*^−/−^ MEFs, while the WT protein efficiently does so. Accordingly, p53^WT^, but not p53^R175H^ and p53^R273H^, increased the sensitivity of *Trp53*^−/−^ MEFs to oxidative stress back to the levels of their *Trp53*^+/+^counterparts.^9^ These data suggest that the cytoplasmic pool of p53 regulates the accumulation of Ca^2+^ ions within the ER, a process that influences the sensitivity of the mitochondrial network to the induction of apoptosis.

Next, Giorgi and colleagues set out to investigate the molecular mechanisms by which cytoplasmic p53 influences reticular Ca^2+^ homeostasis. Pull-down assays in human non-small cell lung carcinoma H1299 cells engineered to overexpress p53 as well as co-immunoprecipitation experiments in *Trp53*^+/+^ MEFs revealed that p53^WT^, but not p53^R175H^ and p53^R273H^, physically binds to SERCA, an interaction that relies on the C-terminal fragment of p53 (aa 294-393).^9^ This domain of p53 is known to accommodate several post-translational modifications,^18^ which, at least theoretically, can modulate its ability to bind (and hence regulate the activity of) SERCA. However, the C-terminal fragment of p53 was unable to influence reticular Ca^2+^ homeostasis and sensitivity to oxidative stress *per se*, indicating that this function resides in another domain of the protein. Of note, the overexpression of SERCA was sufficient to rescue the sensitivity of *Trp53*^−/−^ MEFs to hydrogen peroxide.^9^ This is in agreement with the hypothesis that SERCA operates downstream of p53 in the cascade of events that connects oxidative stress to apoptosis, although it does not formally exclude that these proteins operate independently from each other. Finally, Giorgi *et al.* checked whether p53 would modulate the activity of SERCA by altering its oxidation status. Indeed, p53^WT^ turned out to respond to doxorubicin by limiting the inhibitory sulfenylation of cysteine residues in SERCA, an activity that was not displayed by p53^R273H^.^9^ Thus, cytosolic p53 influences reticular Ca^2+^ homeostasis by regulating the pump activity of SERCA.

To test the relevance of their findings *in vivo*, Giorgi and collaborators developed a novel technological platform for the intravital imaging of Ca^2+^ waves, based on skinfold chambers and the ratiometric Ca^2+^ probe Fura-2.^8^ Using this approach, Giorgi *et al.* were able to monitor Ca^2+^ waves elicited by photodynamic therapy (PDT), an anticancer regimen relying on the administration of an ER-targeted photosensitizer coupled to the exposure of neoplastic lesions to visible light (which promotes oxidative stress), in tumor masses developing *s.c.* in mice. In particular, they tested the ability of neoplastic lesions formed by HRAS^G12V^-expressing *Trp53*^+/+^ or *Trp53*^−/−^ MEFs to respond to PDT by generating Ca^2+^ fluxes that ignite the intrinsic pathway of apoptosis. Confirming their *in vitro* observations, the authors found that *Trp53*^+/+^, but not *Trp53*^−/−^, tumors respond to PDT by accumulating Ca^2+^ ions within the mitochondrial matrix and, as a consequence, initiate the apoptotic program. Moreover, they confirmed that the overexpression of SERCA rescue the sensitivity of *Trp53*^−/−^ tumors to PDT-elicited oxidative stress, as does the overexpression of the mitochondrial calcium uniporter (MCU),^8^ the protein that is responsible for the uptake of cytosolic Ca^2+^ by mitochondria.^19^ Finally, they demonstrated that intercepting intracellular Ca^2+^ ions with the cell-permeant chelator BAPTA-AM significantly reduces the sensitivity of *Trp53*^+/+^ cancers to PDT.^8^ Taken together, these data indicate that the regulation of reticular Ca^2+^ homeostasis by p53 determines the response of established neoplasms to clinically employed inducers of oxidative stress.

The recent papers from Paolo Pinton's laboratory add yet another entry to the ever growing list of p53 functions, the direct control of reticular Ca^2+^ homeostasis. However, several questions remain to be addressed. First, which domain of p53 is responsible for this functional effect (and not just for the interaction between p53 and SERCA)? Second, do compounds that transcriptionally reactivate mutant p53 variants, such as thiosemicarbazone derivatives,^20^ also restore its ability to activate SERCA? Third, what is the role of anti-apoptotic members of the Bcl-2 protein family, which (at least in part) localize to the ER and modulate Ca^2+^ homeostasis, in this process? Shedding light on these and other incognita may drive the development of novel strategies for resensitizing p53-deficient tumors to therapy based on the restoration of Ca^2+^ fluxes. Now, also p53 surfs the Ca^2+^ wave.

## Figures and Tables

**Figure 1:**
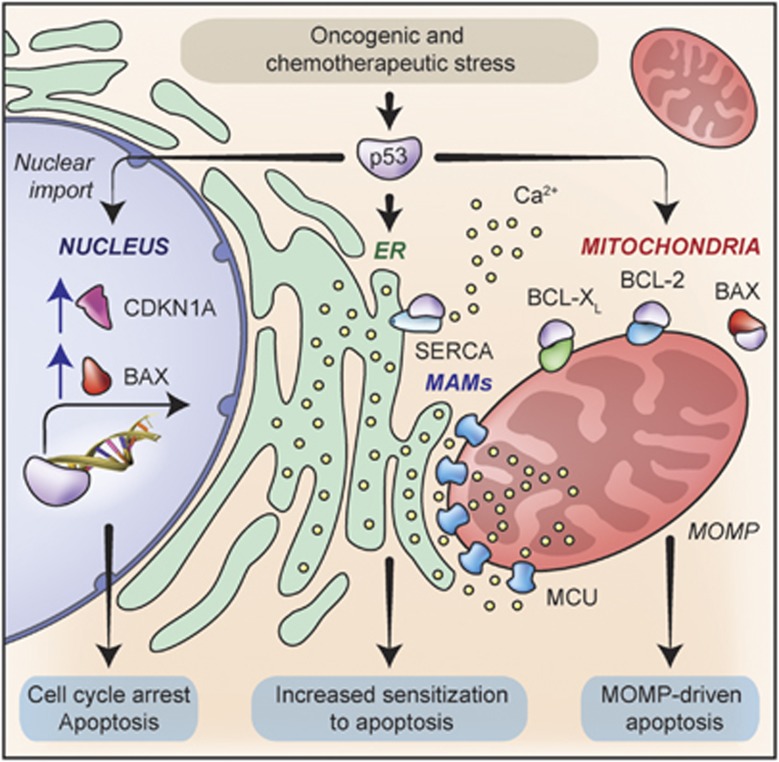
Oncosuppressive functions of p53. In physiological conditions, p53 regulates the expression of several enzymes involved in bioenergetic metabolism and redox balance, hence preserving intracellular homeostasis (not shown). Moreover, p53 respond to various stimuli, including oncogenic stress as well as chemo- and radiotherapy, by orchestrating a cell-wide oncosuppressive program with transcriptional and non-transcriptional branches. In particular, when homeostasis cannot be restored, p53 (1) transactivates several genes coding for cell cycle-arresting factors (e.g., CDKN1A) and proapoptotic proteins (e.g., BAX); (2) physically bind to distinct members of the Bcl-2 protein family at mitochondria (including BAX, BCL-2 and BCL-X_L_), hence promoting mitochondrial outer membrane permeabilization (MOMP)-driven apoptosis; and (3) physically interacts with SERCA at the endoplasmic reticulum (ER), hence facilitating the accumulation of Ca^2+^ ions within the ER lumen. Such an increase in reticular Ca^2+^ concentrations exacerbates pro-apoptotic Ca^2+^ waves elicited in the course of adaptive stress responses, hence reducing the resistance of cells to several perturbations of homeostasis. MCU, mitochondrial calcium uniporter; MAMs, mitochondria-associated ER membranes
